# Current status and advances of fish vaccines in Malaysia

**DOI:** 10.14202/vetworld.2022.465-482

**Published:** 2022-02-26

**Authors:** Mohd Syafiq Mohammad Ridzuan, Azila Abdullah, Rimatulhana Ramly, Nur Nazifah Mansor, Norazsida Ramli, Mohd. Firdaus-Nawi

**Affiliations:** 1 Department of Marine Science, Kulliyyah of Science, International Islamic University Malaysia, Bandar Indera Mahkota, 25200 Kuantan, Pahang, Malaysia; 2 National Fish Health Research Division, Fisheries Research Institute Batu Maung, Department of Fisheries Malaysia, 11960 Batu Maung, Penang, Malaysia; 3 Kulliyyah of Allied Health Science, International Islamic University Malaysia, Bandar Indera Mahkota, 25200 Kuantan, Pahang, Malaysia; 4 Laboratory of Aquatic Animal Health, Institute of Oceanography and Maritime Studies, International Islamic University Malaysia, Cherok Paloh, 26060 Kuantan, Pahang, Malaysia

**Keywords:** aquaculture, fish, fish diseases, Malaysia, vaccine

## Abstract

Fish diseases have a significant negative influence on the Malaysian aquaculture industry. Since the 1980s, the sector has grown in size, which has resulted in a rise in the prevalence of infectious outbreaks affecting both freshwater and marine cultured fish species. Demand for commercially available fish vaccinations is predicted to increase as infectious disease outbreaks continue to occur. In Malaysia, aquaculture vaccine research and development (R&D) are still in its infancy, with most efforts concentrating on producing vaccines against bacterial infections, most notably streptococcosis, vibriosis, and motile Aeromonas septicemia. Despite several attempts, no homegrown vaccine has been effectively introduced into the manufacturing pipeline to date. At the moment, only three imported aquatic vaccines have received full permission, a far cry from the 314 and 60 vaccines licensed in the poultry and porcine industries, respectively. This review will describe recent findings regarding the development of aquaculture vaccines for certain fish species and diseases in Malaysia. In our opinion, R&D on fish vaccines is critical to the aquaculture industry’s viability.

## Introduction

Malaysia has recently developed aquaculture facilities for at least 49 marine and freshwater fish species. The most cultivated marine fish species are Asian seabass, snapper, and grouper. In contrast, the most cultured freshwater fish species are catfish, tilapia, and riverine catfish, with cumulative production of 121,553.75 metric tonnes in 2020 [1]. Numerous culture systems, including ponds, cages, ex-mining pools, cement, and canvas tanks, have been adopted and practiced throughout Malaysia’s coastal and freshwater locations. The fish farming business is developing with continued government backing and favorable response, resulting in intensification measures.

Unfortunately, the sector has also seen a variety of fish diseases in numerous fish species, resulting in massive economic losses and jeopardizing the company’s long-term viability. Current solutions rely on a combination of good farm management and the use of commercially accessible antibiotics. The Malaysian government recently developed a national action plan on antimicrobial resistance (MyAP-AMR) for the period of 2017-2021, which discourages the abuse of antibiotics in the food production industry. Furthermore, different efforts have been made to raise national awareness about AMR and antibiotics, expand the information and evidence base through surveillance and research, and optimize antimicrobial drug use [2]. Vaccination and alternative medicine are considered as a way out under these circumstances and have grown in popularity among local researchers, although acceptance among farmers remains questionable.

Several advancements and notable accomplishments have been made locally in the development of aquaculture vaccines against fish diseases. As a result, the purpose of this review is to offer an update on recent aquaculture vaccine development in Malaysia for major fish species and diseases. We genuinely hope that this analysis provides insight into the future potential for aquaculture fish vaccine development.

## General Overview of Recent Aquaculture Status in Malaysia

The fisheries sector provides a significant portion of income for more than 106,000 local fishermen and farmers in Malaysia [1]. In addition, fisheries provide up to 12% of agriculture’s gross domestic product (GDP) and 0.9% of the country’s GDP [3]. While it may seem insignificant, the fisheries sector is vital in supplying food, jobs, nutrition, and a healthy lifestyle and supporting other downstream businesses. Malaysia’s capture fisheries and aquaculture production totaled 1.6 million tonnes in 2020, a decrease of 2.36% from 2019. Table-1 [1,3,4] compares the overall fisheries productivity in Malaysia from 2016 to 2020.

**Table 1:** Fisheries and aquaculture production between 2016 and 2020 in Malaysia [1,3,4].

Year	2016	2017	2018	2019	2020

	(Tonnes, live weight)				
Production					
Capture					
Inland	5847.97	5177.19	6089.08	5568.70	5625.14
Marine	1,574,447	1,465,113	1,452,862	1,455,446	1,383,299
Total capture	1,580,295.0	1,470,290.2	1,458,951.1	1,461,014.7	1,388,924.1
Aquaculture*^1^*					
Inland	103,348.21	102,596.83	101,269.88	104,601.56	97,209.74
Marine	98,049.9	121,453.02	116,112.08	119,069.47	120,739.51
Total aquaculture	201,398.11	224,049.85	217,381.96	223,671.03	217,949.25
Total fisheries and aquaculture*^2^*	1,781,693.1	1,694,340.0	1,676,333.0	1,684,685.7	1,606,873.4

1 Exclude production of seaweed.

2 Total may not match due to rounding

Malaysia’s aquaculture sector is thriving with huge potential and ongoing government support to supply the rising demand for animal protein. Aquaculture is critical for boosting fish production, balancing demand on capture fisheries, and preventing wild fish overexploitation. Globally, aquaculture currently accounts for 46% of total fish production [5], with Asia accounting for more than 91% of worldwide aquaculture production [6]. In 2020, Malaysia had a total of 20,262 fish farmers. About 73.5% (7,349 farmers) were engaged in freshwater aquaculture, while 16% (3,349 farmers) were engaged in brackish water aquaculture. In contrast, the remainder were in other sub-sectors classified as ornamental fish, aquatic plants, or seaweed. Malaysian freshwater aquaculture production totaled 97,209.74 tonnes worth RM 766 million in 2020, while brackish aquaculture production totaled 120,739.51 tonnes worth RM 2,289.39 million (Table-1).

On the other hand, seaweed production was estimated to reach 182,061.00 tonnes of wet weight in 2020, accounting for RM 58.87 million of the wholesale value. Malaysia’s aquaculture fish production system relies on conventional technologies such as ponds, ex-mining pools, cages, tanks, and pen culture (Table-2) [1]. The utilization of cutting-edge technology such as recirculating aquaculture systems is currently restricted to government hatcheries.

**Table 2:** Fractions of aquaculture production systems and areas adopted in Malaysia [1].

Production system/area	Pond (Ha)	Ex-mining pool (Ha)	Cages (m^2^×10^3^)	Tank^1^ (m^2^×10^3^)	Pen culture (Ha)	Molluscs culture^2^ (Ha)	Seaweed culture (Ha)
Freshwater	3725.29	3033.00	550.00	417.99	8.00	-	-
Brackish water	7511.00	-	2304.47	239.00	392,368.00	9714.00	9828.00
Total	11,236.29	3033.00	2854.47	656.99	392,376.00	9714.00	9828.00

1 Include cement and canvas tank.

2 Include cockle (bottom culture), mussel, and oyster (raft culture)

## Infectious Diseases of Fish in Malaysia

Infectious diseases are any abnormalities caused by a wide range of microorganism infections. Bacteria and viruses are the main culprits in Malaysia’s outbreaks of infectious illnesses in the aquaculture industry [7]. The most common bacterial diseases that affect cultured fish are *Streptococcus agalactiae*, *Streptococcus iniae*, *Aeromonas hydrophila*, and *Vibrio alginolyticus* [8-10]. Meanwhile, betanodavirus and iridovirus frequently affect cultured marine fish species, and recently the outbreaks of emerging diseases of tilapia lake virus (TiLV) were reported to affect the wild [11] and cultured tilapia [12]. These diseases have contributed substantial economic losses to the country, and actions must reduce the impact. Vaccination was considered the best control measure to protect the fish from infectious diseases by developing herd immunity [13]. A recent study by Standish *et al*. [14] proved the existence of herd immunity in aquatic animals, which played a crucial role in increasing the survival rate and disease resistance.

## Overview of Fish Vaccines Licensing in Malaysia

**Figure-1:**
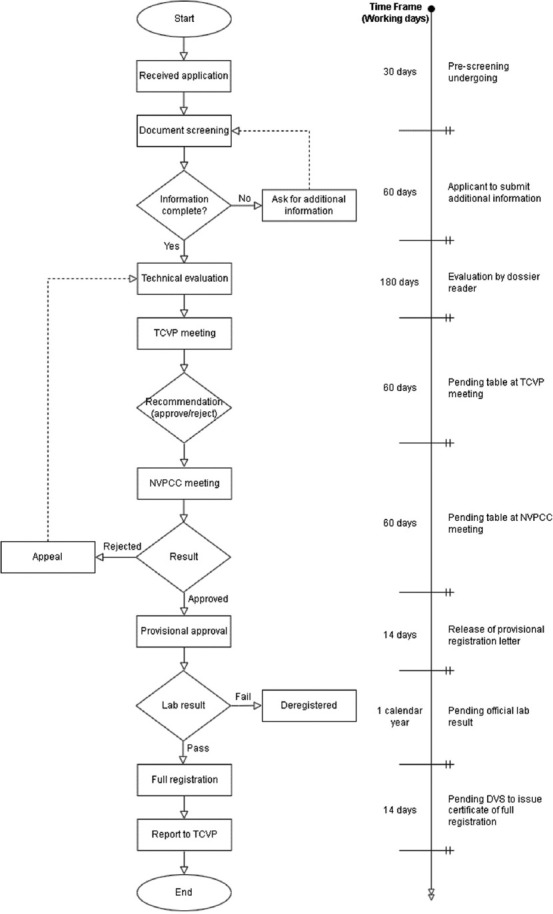
Flow chart and time frame of veterinary vaccines registration in Malaysia [15].

The approval of any veterinary vaccine registration in Malaysia, including fish vaccines, is governed by the Department of Veterinary Services (DVS), Ministry of Agriculture and Food Industry, and is governed by the Animal Act 1953. As stated in Section 30(1) of the Animal Act 1953 (revised in 2006), the Director-General of DVS has the authority to provide a license authorizing the holder to possess live cultures or veterinary vaccinations and to administer them to animals or birds. In addition, Section 84(1) of the Animal Act 1953 (Amendment 2013) stated that no person should knowingly import or possess any living noxious insect, pest, or living germ, virus, or bacterial culture, that is harmful or dangerous to animals or birds without the Director-prior General’s written permission. Therefore, the DVS as a regulatory agency controls the production, importation, distribution, sale, and use of veterinary vaccines for diagnosis, treatment, research, and disease prevention. The Technical Committee of Veterinary Product and National Veterinary Product Control Committee under DVS act as a licensing authority to register animal vaccines and other biologics. The licensure of new animal vaccines is subjected to a well-defined regulatory process. A general framework for the regulatory approval of veterinary vaccines is shown in Figure-1 [15].

At present, Malaysia has approved only three aquatic vaccines, which are shown in Table-3 [16]. These vaccines are inactivated and are meant to protect against streptococcosis, vibriosis, and iridoviral disease. Notably, other vaccine forms, such as recombinant, DNA, or live-attenuated vaccinations, have not been licensed for aquaculture use in Malaysia.

**Table 3:** List of approved aquatic vaccines in Malaysia [16].

Trade name	Causative agent	Manufacturer	Nature of vaccine	Recommended species	Delivery methods*^1^*	Dose and recommended fish size	Further information
AquaVac Strep SI	*S. iniae*	Intervet, Holland	Killed	Tilapia; Asian seabass; Other susceptible fish species	IP; IMM	Injection: 0.1 mL/fish; for fish 20 g or above Immersion: 1 L of vaccine and 9 L of seawater; for fish 3 g and above	www.aquavac-vaccines.com/
AquaVac IridoV	Iridovirus	Intervet, Holland	Killed	Asian seabass; Grouper; Pompano; Japanese yellowtail	IP	0.05 mL/fish; for fish 5 g or above	www.aquavac-vaccines.com/
Vibri- Fishvax	*Vibrio anguillarum*	Fatro S.p.A, Italy	Killed	Trout; Salmon; Seabass; Seabream	IP; IMM	Injection: 0.1 mL/fish; for fish 50 g or above Immersion: dip 7.5-10 kg of fish per 1 L of vaccine (1:10 ratio); fish between 1 and 8 g	www.fatro.it

1 IP=Intraperitoneal injection, IMM=Immersion

## Overview of Recent Research and Development (R&D) of the Fish Vaccine in Malaysia

Malaysian research on fish vaccines predates 1979 and has lagged much behind other countries. Initially, this project was sparked by the rapid expansion of marine fish mariculture, including sea bass (*Lates calcarifer*), grouper (*Epinephelus coioides*), and golden snapper (*Lutjanus johni*), which began in 1973 and accelerated in the 1980s. Fingerlings or juveniles were brought in large quantities from neighboring countries such as Chinese Taipei, Indonesia, and Thailand to deal with vast demand and concentration [17]. Due to the increased intensity of aquaculture, an episode of disease outbreak is frequently experienced during the initial stocking and grow-out stage. Throughout a 1-year survey conducted by Chuah [18], it was discovered that groupers were primarily linked to viral diseases (50%), with bacterial infections accounting for 47% and cryptocaryoniasis accounting for the remaining 3%. Based on the culturists’ descriptions of the discoloration (blackening) of the fish body without other external symptoms, it was suspected that the virus-associated disease was iridoviral infection, whereas the bacterial-associated disease of *Vibrio* spp. and *Flexibacter* spp. caused ulceration or rotting of the groupers’ fins and tail.

One of the earliest studies on the fish vaccine in Malaysia was recorded in the late seventies. See-Yong *et al*. [19] explored the root cause of red boil disease in estuary grouper (*Epinephelus salmoides*) with the possibility of vaccination control. Their study found that *Vibrio parahaemolyticus* is the cause of disease based on colony morphology on selective Thiosulfate-citrate-bile salts-sucrose media and pathogenicity test. Unfortunately, they were unable to develop effective vaccines against *V. parahaemolyticus* utilizing several means of inactivation, including heat-killed and formalin-killed, with and without adjuvant. When challenged 1 week after immunization, all the vaccines failed to protect the fish, with 60-70% mortality rates. However, the absence of protection was most likely caused by the bacterium being challenged before the immunity developed.

Another mass vaccination trial was conducted in Malaysia in the late 1990s, with special permission granted to a government research institute, the National Fish Health Research Centre (NaFisH), Fisheries Research Institute Batu Maung, Penang, to conduct a field vaccination trial using Alpharma vaccine on *Lutjanus argentimaculatus* against vibriosis [20]. A total of 19,000 fish were vaccinated in-house at the hatchery before being sent to Langkawi Island for cage culture. Unfortunately, between 24 and 48 h after immunization aberrant mortality (100%) was seen, along with bulging of the ocular membrane on the dead fish. As a result, a replicated study on a laboratory scale was done to ascertain any predisposing conditions that may have contributed to the situation. Initially, it was suspected that the vaccine or its storage conditions were to blame. However, the vaccine had no detrimental effects, and no abnormalities were observed during the laboratory testing. In addition, the physical and behavioral alterations observed within 24-48 h post-vaccination were missing after 2 weeks of monitoring, as described in the field trial. As a result, they hypothesized that prior catastrophic incidents could have been caused by handling stress and a high stocking density in the holding net prior to injection. Since then, local researchers have made enormous strides, as seen by an increase in the volume of study on epidemiology, pathogenicity, vaccine development, and other relevant topics. This section will discuss the state of fish vaccine R&D in Malaysia for specific fish species and diseases.

### Streptococcosis vaccine

Streptococcosis is one of the most extensive vaccines studied in Malaysia due to the aggressive series of outbreaks affecting tilapia culture in Malaysia, dating back to 1997 [21,22]. The disease is a bacterial infection caused by *Streptococcus* species. *S. agalactiae* and *S. iniae* are the major species affecting tilapia aquaculture production worldwide, including Malaysia [8,23,24]. The clinical symptoms and signs of streptococcosis infected fish include exophthalmia or pop-eye, corneal opacity, hemorrhage at the gill and body surface, inflammation and soft brain, ascites, and display erratic swimming behavior [24,25]. Histological examination of hybrid tilapia naturally infected by *S. agalactiae* revealed marked congestion and infiltration of inflammatory cells in the eye, brain, kidney, liver, and spleen [26].

The first reported case of streptococcosis in Malaysia was documented in the late 1990s at Pahang River, Pahang affecting tilapia weighing between 300 and 400 g and resulting in 60% mortality of cultured red hybrid tilapia. In 2000, a series of disease outbreaks were recorded in Kenyir Lake, Terengganu, and Pergau Lake, Kelantan, resulting in approximately 50% mortality of cultured tilapia [21,22,27]. Repeated cases were encountered in Aquaculture Industrial Zone at Kenyir Lake [28] and Temerloh, Pahang [26], causing mass mortality of cage-cultured red hybrid tilapia. The outbreaks commonly occur during hot and dry months and are correlated with high water temperature (≥29°C) [29]. A similar finding by Pei-Chih *et al*. [23] reported that water temperature above 27°C was predisposed tilapia to streptococcosis infection in Taiwan. Furthermore, in an experimental trial, Rodkhum *et al*. [30] showed Nile tilapia that were exposed to a high water temperature of 30°C and 33°C, after immersion challenged with *S. agalactiae*, resulted in high cumulated mortality compared to the tilapia maintained at 25°C where most of the fish survived without any clinical signs.

More recently, Siti Hawa *et al*. [8] reported that *S. iniae* was responsible for some of the cases in cultured red tilapia since 2006. Currently, reported disease cases are widespread, covering all over Peninsular Malaysia. An extensive review of the status of the disease in Malaysia was previously described by Zamri-Saad *et al*. [31]. Although the evidence of *S. agalactiae* serotype Ia was recently discovered [32], local isolates predominantly corresponded to Serotype III and Biotype 1 [33]. In Malaysia, infection by *S. agalactiae* has also been reported in golden pompano, *Trachinotus blochii* [34].

An epidemic of streptococcosis outbreaks was also documented worldwide. For instance, in his recent article, Pei-Chih *et al*. [23] reported that the clinical cases of *Oreochromis* spp. in Taiwan were predominated by bacterial infection, where streptococcosis accounted for 53.7% of the cases, and the infection rate was recorded at 29.5%. Meanwhile, in China, sporadic cases of streptococcosis involving *S. agalactiae* have been reported in several provinces of Guangdong, Guangxi, Hainan, and Fujian, which led to massive cumulative mortality and economic losses [35]. It is worth noting that these four central tilapia-producing provinces accounted for 40% of global tilapia production. In addition, between 2016 and 2017, six disease outbreaks were reported in Brazil, affecting Nile tilapia with total death rates of 25-35% [36]. Further investigation revealed that the primary etiological agent was *S. agalactiae* Serotype III. Recently, the disease was reported to have caused enormous losses in Thailand’s freshwater farmed seabass, with a cumulative mortality rate of 35-65% [37]. Interestingly, Al-Harbi [38] previously reported evidence of non-hemolytic Group B *S. agalactiae* infection resulting in typical streptococcal clinical symptoms, culminating in acute or chronic infections of cultured tilapia in Saudi Arabia. Streptococci-related global losses were previously estimated at USD250 million in 2008 [39,40].

Hence, it is now realized that these bacteria pose a considerable threat to tilapia farming, so producing an effective vaccine is rather urgent. In Malaysia, several types of vaccines, such as inactivated, recombinant, and live attenuated vaccines; Different routes of vaccination, such as oral, injectable, or immersion, were extensively explored by local researchers against *S. agalactiae* and *S. iniae* (Table-4) [41-59].

#### Development of a vaccine against S. agalactiae

Rather than developing an injectable or immersion vaccine against streptococcosis, significant resources were expended in developing an oral vaccine. The oral route becomes the most preferred route because 80% of the local tilapia culturists are small-scale operators. Utilizing injectable or immersion courses necessitated additional infrastructure and labor, a massive endeavor that added to the load on these farmers. Oral vaccines, which are generally prepared by infusing the antigen into feed, are suited for mass vaccination of fish of all sizes and ages. Oral administration is the most appealing way since it is straightforward, less stressful for fish, and provides a more flexible approach to vaccination regime formulation [60].

Despite offering attractive features, it is essential to note that oral vaccination also has its limitations and usually provides a shorter duration of protection when compared to injection immunization. Furthermore, oral administration makes it impossible to determine the exact dose of antigen each fish takes and occasionally results in inconsistent responses because of the fish’s harsh acidic stomach contact. As a result, the antigen is susceptible to being destroyed prior to rousing the gut-associated lymphoid tissues (GALTs) and priming immune cells [61]. Therefore, conjugation with a large immunogenic carrier called an adjuvant is crucial to permit a prolonged antigen release [62]. Different types of adjuvants have been tested for fish vaccines, such as alginate microspheres encapsulated [63], nanoparticle [64], Freund’s incomplete adjuvant (FIA) [65], and polysaccharides [66], with promising results.

The effect of oral vaccination of killed whole-cell *S. agalactiae* vaccine on stimulating GALTs in tilapia was previously investigated by Firdaus-Nawi *et al*. [67]. Their study discovered that once-weekly vaccination exposure was adequate to stimulate the GALT, but repeated doses elicited stronger responses. Moreover, the size of GALT and the number of aggregated lymphoid cells in tilapia’s gastrointestinal tract were much greater than in unvaccinated fish. Their finding provides an early indication that oral exposures to the killed antigen incorporated in feed could be used in a vaccination procedure to protect tilapia from *S. agalactiae* infection.

The protective capacity of locally produced oil adjuvanted oral vaccine against streptococcosis in tilapia was subsequently reported [41]. They investigated the effect of immunological response between fish fed with adjuvanted feed-based vaccine, feed-based vaccine (without adjuvant), and unvaccinated groups, following a vaccination regime in a laboratory vaccination trial. They found that following the vaccination regime, the antibody level (immunoglobulin M [IgM]) in serum, mucus, and gut lavage fluid of both vaccinated groups was significantly higher (p<0.05) as early as 1-week post-immunization, and the increasing pattern was constantly observed till the end of the experiment of week 7. Moreover, the antibody level produced in fish fed with the adjuvanted feed-based vaccine was also significantly higher (p<0.05) than fish provided with the feed-based vaccine (without adjuvant). Intraperitoneal challenge against high concentration of live virulent *S. agalactiae* resulted in 100% survival in fish fed with adjuvanted vaccine group, while 50% and 12.5% survival of fish fed with the feed-based vaccine (without adjuvant) and control group, respectively. Another laboratory trial of a feed-based vaccine against *S. agalactiae* in red tilapia showed that a double booster vaccination regime (weeks 0, 2, and 6) at 4% of body weight successfully provided a consistently high level of serum IgM antibody for 4 months [42]. Following the excellent results in the laboratory setting, a field trial was undertaken and tested in an endemic farm [43]. A similar vaccination regime was employed in the field study and resulted in 75% survival of the vaccinated group compared to 45% survival in the unvaccinated group. Interestingly, the author and coworkers demonstrated a comparable serum antibody (IgM) pattern between laboratory and field trials.

A more advanced approach in antigen preparation of *S. agalactiae* involving recombinant DNA technology such as insertion of genetic materials that encode antigen into an expressing vector was also explored by local researchers. As such, Nur-Nazifah *et al*. [44] previously reported that an adequate protective capacity of an inactivated recombinant vaccine expresses the cell wall surface anchor family protein of *S. agalactiae* in tilapia, following high dose challenge in a heat-stress environment with a record of 70% survival. Again, an elevated antibody level in blood serum, body mucus, and gut lavage fluid was significantly higher (p<0.05) and correlated with protection rate. Thus, they believed that the application of appropriate adjuvant could tremendously enhance vaccine efficacy. This finding has led to the vaccination field trials carried out in Kenyir Lake, Terengganu, and East Coast of Malaysia [45]. The same recombinant vaccine that expresses the cell wall surface anchor family protein of *S. agalactiae* in the presence of FIA was used and administered orally during both rainy and dry seasons. In both seasons, the vaccinated group showed elevated levels of serum, mucus, and gut lavage fluid antibody (IgM) (p<0.05) as early as 2^nd^ month post-vaccination and continued to increase after booster (given at month-2) until the end of the trial at 4^th^ month. Stimulation of the GALT was also observed after the 1^st^ month of antigen exposure in the intestine as individual cells or small aggregations in both the epithelium and lamina propria. Better stimulation of GALT was found at the 4^th^ month correlated with repeated exposure of antigen of a booster dose.

More recently, Sa’aidatun Asyikin *et al*. [46] reported the utilization of palm oil as an adjuvant substitute in *S. agalactiae* oral vaccine with promising results. Palm oil is unique among vegetable oils because of its high saturated fatty acid content at the 2-position of its triglycerides and is an excellent source of cheaper adjuvant alternatives due to the vitamin E and saponin content. Both have previously been shown to enhance cell-mediated immunity and antibody production [46,68]. In addition, another work by Mufti *et al*. [69] showed that a mixture of palm and coconut oil adjuvants in a duck pasteurellosis nanovaccine was physicochemically stable following a 6-month pretreatment period at 4, 25, and 40°C.

Another route of administration of vaccine against the disease was also extensively explored. For example, Noraini *et al*. [47] reported the potential use of spray administration of the formalin-killed *S. agalactiae* vaccine earlier. Antigen administration through spray could theoretically transfer antigens to mucosal tissues, such as the skin, gill, and nasal, where they can activate innate and adaptive immune responses in the fish [70]. The authors evaluated the frequency effect of spray vaccination on fish that received a single spray, fish that received a daily spray for 5 consecutive days, and fish that were not vaccinated. In the 2^nd^ and 4^th^ weeks, each group received two boosts. Serological analysis revealed that both serum and mucus IgM levels of vaccinated fish were significantly higher (p<0.05) after the 1^st^ week of post-vaccination and after each booster compared to control. The challenge trial against *S. agalactiae* at a 10^9^ CFU/mL concentration using both immersion and intraperitoneal (i.p) methods resulted in 80% and 70% survival rates, respectively. These findings indicated that spray administration effectively generated protective IgM antibodies providing moderate to high protection.

Another study by Kahieshesfandiari *et al*. [48] investigated the potential use of feed-based heat-inactivated biofilm vaccine of *S. agalactiae*. Chitin flakes were used to encapsulate the biofilm cells, and they compared the efficacy of the biofilm vaccine against the conventional heat-killed whole-cell vaccine in red hybrid tilapia. The vaccination regime was standardized between both types of vaccines. The vaccines were administered on week 0 and a booster on week two before the fish were subjected to the i.p challenge of a high concentration of *S. agalactiae* (10^9^ CFU/mL). The results showed that tilapia vaccinated with biofilm vaccine exhibited significantly robust immune response (p<0.05) and high protection with the relative percent survival (RPS) of 87% as compared to fish vaccinated with the whole-cell vaccine (57%). Besides that, they said that fish immunized with biofilm vaccine had significantly higher serum, mucus, and gut lavage antibody levels (p<0.05) than fish inoculated with the whole-cell vaccine or the control group. Furthermore, they discussed evidence for a high level of GALT activation in the form of lymphoid cell aggregations within the lamina propria.

Furthermore, continuous effort to develop an efficacious vaccine against streptococcosis has led the local researcher to investigate the potential application of a live-attenuated vaccine, another modern type of vaccine whereby the bacteria hold weakened or less virulent forms. Laith *et al*. [49] previously demonstrated that repeated 187 laboratory passage and chemical attenuation using acriflavine dye could weaken *S. agalactiae*. Primary oral administration of live-attenuated vaccine and booster at week 4 resulted in RPS of 82% after i.p challenge with a median lethal dose (*LD_50_*) of *S. agalactiae*. In addition, significant enhancements on the immune responses of tilapia were described, including elevated serum antibody (IgM) level and lysozyme activity.

#### Development of a vaccine against S. iniae

R&D of an oral vaccine against *S. iniae* was also undertaken by local researchers. Evidence of antigen localization in the gut of oral vaccinated and non-vaccinated red hybrid tilapia post-challenge with *S. iniae* was recently described by Hayat *et al*. [50]. A low number of antigens at 72 h post-challenge in the group receiving the routine oral vaccine (5 consecutive days and booster on days 14 and 21) displayed the simulation and irregular projection of M-cells with variable morphology and antigen distribution pattern. Further, they reported that fish immunized with biofilm vaccine had significantly higher antibody levels in their serum, mucus, and gut lavage (p<0.05) than fish immunized with the whole-cell vaccine or the control group [51]. Moreover, they discussed evidence indicating a high level of GALT stimulation in the lamina propria in the form of lymphoid cell aggregations.

### Motile aeromonas septicemia (MAS) vaccine

Infection with aeromonad bacteria causes MAS, which is most usually associated with *A. hydrophila* [71,72], but other species such as *A. sobria* [71,73], *A. veronii* [74,75], and *A. caviae* [76] have been documented to have a similar etiology in the past. The *Aeromonas* genus consists of many species. They are all motile, except for *A. salmonicida*, and most of them are ubiquitous inhabitants of different aquatic ecosystems [77]. The foremost, *A. hydrophila*, is predominantly responsible for most cases of MAS in freshwater fish worldwide. For example, in Malaysia, cases involving *A. hydrophila* have been reported in *Clarias* spp. [78], *Pangasius* spp. [79], and tilapia [80].

Perhaps this disease is sometimes overlooked since the infection’s nature is typically associated with external stressors such as poor water quality or infection by another pathogen. Although the bacterial is often regarded as an opportunistic pathogen, it is easily transmitted from fish to fish, with hemorrhagic and ulcerated skin indicating the severity of infection. Depending on the strain of bacteria, the portal of entry, and mechanism of infection, fish infected with MAS can produce a diverse pattern of skin lesions [81], which indirectly affects the aesthetic quality of fish and market value, particularly in trading live fish. The previous research shows that the combination of various virulence factors might influence the pathogenicity of *Aeromonas* spp. in fish. Some of these virulence factors include exotoxins [82], exoenzymes [83], outer membrane protein (Omp) [84], and quorum sensing-controlled virulence factor [75].

The disease has resulted in severe economic losses to aquaculture. In 2009, an outbreak of *A. hydrophila* was reported in Brazil, affecting farmed hybrid surubim amounting to 20 tonnes in mortality and approximately USD 160,000 losses [85]. Peterman and Posadas [86] reported an estimated farm-gate loss of USD 2.6 million due to the disease within catfish aquaculture in east Mississippi during 2016. Another alarming concern on *Aeromonas* spp. is their increased resistance pattern against multiple antibiotics, especially those currently used in aquaculture [87,88].

Several countries have actively worked to address this issue, and vaccination is viewed as a viable option for preventing the disease. India, for example, has conducted substantial research on different types of vaccines, including inactivated vaccines, live attenuated vaccines, and biofilm vaccines, all of which are based on various components of *A. hydrophila*. Previously, extracellular protein, S-layer protein, lipopolysaccharide, and Omp were identified as viable candidates [89]. Although local researchers in Malaysia are not interested in developing a vaccine against *A. hydrophila*, this is likely due to its complicated serotype and variable in virulence gene expression [70,90]; many efforts have been conducted in recent years and are summarized in Table-4.

**Table 4:** Different experimental approaches and trials in the development of fish vaccine in Malaysia.

Name of pathogen (s)	Type of vaccine	Vaccination route	Stage of trial	Fish species	Finding	Reference
*S. agalactiae*	Inactivated	Oral	Lab trial	Red tilapia	• Adjuvanted vaccine group resulted in the highest survival rate (100%), followed by non-adjuvanted group (50%) and control (12.5%) • Adjuvanted vaccine group resulted in significant higher IgM level, increase in the size of gut-associated lymphoid tissue and number of lymphocytes	[41]
*S. agalactiae*	Inactivated	Oral	Lab trial	Red tilapia	• Double booster group resulted in highest survival (70%), followed by single booster group (45%) and control (0%) • Following first administration and booster dose, both vaccinated groups showed significantly higher serum IgM levels and reached the peak at week-3 before declining. Following the second booster in week 6, the antibody level significantly increased again and remained high until week-12	[42]
*S. agalactiae*	Inactivated	Oral	Field trial	Red tilapia	• The survival rate was 75% for the double booster group, 65% for single booster and 45% for unvaccinated • Following vaccination, both vaccinated groups showed a significant increase in the antibody level that reached the peak at week-4 but gradually declined thereafter. However, following another booster at week-6 (double booster group), the antibody level showed a significant increase and remained high until the end of the study period	[43]
*S. agalactiae*	Recombinant	Oral	Lab trial	Red tilapia	• The survival rate was 70% for recombinant vaccine • Feed-based recombinant vaccine developed a strong and significantly higher IgM antibody response in serum, mucus, and gut lavage fluid	[44]
*S. agalactiae*	Recombinant	Oral	Field trial	Red tilapia	• No outbreak of streptococcosis was recorded during the study period • The IgM antibody level in serum, mucus, and gut lavage was significantly higher in vaccinated groups. Aggregation of lymphocytes and development of GALT was observed in vaccinated fish	[45]
*S. agalactiae*	Inactivated	Oral	Lab trial	Red tilapia	• The highest survival rate (70%) was recorded in the vaccinated group using 10% palm oil, followed by 7% Freund’s incomplete adjuvant (45%) • Both vaccinated groups displayed higher antibody response with a similar pattern	[46]
*S. agalactiae*	Inactivated	Spray immersion	Lab trial	Red tilapia	• A higher percentage of survival was noted for fish challenged through immersion (80%) compared with an intraperitoneal injection (70%) • Serum and mucus antibodies correspond to each booster. Both antibody levels remained high after the last booster until the end of the 8-week study period	[47]
*S. agalactiae*	Inactivated and biofilm	Oral	Lab trial	Red tilapia	• Fish vaccinated with biofilm vaccine showed the highest survival percentage (87%), followed by fish fed with whole-cell vaccine (57%) • The serum, mucus, and gut lavage antibody level of fish vaccinated with biofilm vaccine was significantly higher than other groups	[48]
*S. agalactiae*	Live attenuated	Oral	Lab trial	Red tilapia	• The RPS of fish vaccinated with live attenuated vaccine was 82% compared to 2.5% in control • After the first and booster dose, the serum IgM levels increased significantly and reached the peak on the week-5	[49]
*S. iniae*	Inactivated	Oral	Lab trial	Red tilapia	• No efficacy data provided • Group of fish vaccinated for three and five consecutive days with booster on day-14 and day-21 recorded the lowest antigen positivity • Group of fish vaccinated for five consecutive days with booster on day-14 and day-21 showed the stimulation and irregular projection of M-cells with variable morphology and antigen distribution pattern	[50]
*S. iniae*	Inactivated	Oral	Lab trial	Red tilapia	• Groups of fish vaccinated continuously for 9 days with booster on day-14 and day-21 recorded the highest survival of 70% • All vaccinated groups showed a significant increase in IgM levels in serum, mucus, and gut lavage	[51]
*Aeromonas hydrophila*	Recombinant	Intra-peritoneal	Lab trial	African catfish	• The RPS of all vaccinated groups was significantly higher (100%) compared to placebo (29.42%)	[52]
*S. iniae; A. hydrophila*	Inactivated; bivalent	Oral	Lab trial	Red tilapia	• Group of fish vaccinated by bivalent vaccine incorporated in feed achieved the highest RPS of 80%, 77% and 77% following challenged against *S. iniae, A. hydrophila,* and co-infection of both bacteria, respectively • Lysozyme and phagocytic activity and, serum antibody was significantly higher against *S. iniae and A. hydrophila* in vaccinated groups in the pre and post-challenged	[53]
*V. harveyi; S. agalactiae; A. hydrophila*	Inactivated; Polyvalent	Oral	Lab trial	Asian seabass	• The vaccine provided a RPS of 75%, 80%, and 80% after challenge with *V. harveyi*, *A. hydrophila*, and *S. agalactiae*, respectively • The serum antibody and lysozyme remained significantly higher at the end of 6 weeks trial. The immune-related gene, dendritic cells, Chemokine ligand 4, and major histocompatibility complex class I were highly expression after the fish were vaccinated with the oral vaccine	[54]
*V. harveyi*	Live attenuated	Intra-peritoneal	Lab trial	Tiger grouper	• The RPS of the vaccinated group was calculated at 52% • Vaccinated fish displayed upregulation of autophagosome pathway, the coagulation and complement cascade pathways, and antigen processing and presentation pathways	[55]
*V. harveyi*	Live attenuated	Bath immersion	Lab trial	Asian seabass	• Fish vaccinated with live attenuated vaccine resulted in a significantly high rate of survival (68%) after being challenged with the wild type strain • Increase expressions of the Chemokine ligand 4 and major histocompatibility complex I genes in the skin and liver of the vaccinated fish	[56]
*V. harveyi*	Inactivated	Intra-peritoneal	Lab trial	Marine red tilapia	• Vaccinated group resulted in a significantly higher rate of survival (87%) • The IgM antibody titer and lysozyme of vaccinated fish were significantly higher throughout the experiment	[55]
*V. alginolyticus*	Recombinant	Intra-peritoneal	Lab trial	Hybrid grouper	• The RPS for the rOmpK vaccinated group was 100%, followed by 63% for the rOmpW group • Both rOmpK and rOmpW vaccinated groups displayed an increasing pattern of serum IgM following vaccinations • The IgM against rOmpK showed significantly higher values than rOmpW at the challenge on day 28 post-vaccination	[57,58]
*V. alginolyticus; V. harveyi*	Recombinant; bivalent	Intra-peritoneal	Lab trial	Asian seabass	• Fish vaccinated with r-OmpK had a 90% survival rate against *V. harveyi* and 100% against *V. alginolyticus* and *Vibrio parahaemolyticus* • The blood and gut lavage antibody of fish vaccinated with r-OmpK were increased significantly and TLR2, MyD88, and MHCI genes were upregulation in the kidney and intestinal tissues of r-OmpK vaccinated fish	[59]

*S. iniae=Streptococcus iniae, A. hydrophila=Aeromonas hydrophila, V. harveyi=Vibrio harveyi, A. hydrophila=Aeromonas hydrophila, S. agalactiae=Streptococcus agalactiae, V. alginolyticus=Vibrio alginolyticus*, RPS=Relative percent survival

#### Development of a vaccine against A. hydrophila

Matusin [52] reported her successful attempt in developing a recombinant vaccine expressing the immunogenic genes of Omp (OmpTs and OmpW) of *A. hydrophila*. The genes were positively cloned into the pET102/D-TOPO vector, and trials were conducted in African catfish (*Clarias gariephinus*). Two vaccines were administered by injection at week-0 followed by a booster at week-2 before being challenged with live virulent *A. hydrophila*. The RPS of vaccinated groups was recorded at 100% compared to the placebo group (29.42%).

In contrast, Monir *et al*. [53] previously reported the effectiveness of a newly developed feed-based bivalent vaccine against *S. iniae* and *A. hydrophila* in combating both pathogens in tilapia. They discovered that following double booster vaccination regime (given on 0, 14, and 42 days), the number of leucocytes, monocytes, granulocytes, lysozyme, and phagocytic activity, and serum antibody (IgM) levels were significantly higher (p<0.05) in the vaccinated group as early as 21 days post-vaccination. In addition, the challenge test against *S. iniae*, *A. hydrophila*, and co-infection of these bacteria showed the RPS of 80%, 76.67%, and 76.67%, respectively.

More recently, another study by Aslah *et al*. [54] reported the effectiveness of a newly developed feed-based polyvalent vaccine against *Vibrio harveyi*, *S. agalactiae*, and *A. hydrophila* in Asian seabass. This appealing feature effectively controls several fish diseases by a single vaccination. They found that oral vaccination at 5% body weight has successfully stimulated high serum antibody (IgM) production (p<0.05) against *V. harveyi*, *A. hydrophila*, and *S. agalactiae*, and serum lysozyme level. Gene expression analysis from vaccinated fish gut samples revealed significantly higher expression (p<0.05) of dendritic cells, complement-3, chemokine ligand 4, and major histocompatibility complex class I (MHC I) compared to the unvaccinated group. The experimental challenge resulted in RPS of 75%, 80%, and 80% after i.p injection with 10^7^ CFU/mL of *V. harveyi*, *A. hydrophila*, and *S. agalactiae*, respectively.

### Vibriosis vaccine

Vibriosis has been an issue in Malaysian marine aquaculture since 1973 [17], when the culture of marine finfish was established. However, it was not until the 1990s that sea bass, grouper, and snapper were frequently reported to be infected with the disease. Throughout the hatchery and grow-out phases, the fish are susceptible. Several *Vibrio* spp. have been discriminated against as etiological agents of the disease, including *V. alginolyticus*, *Vibrio vulnificus*, and *Vibrio harveyi*. In 1979, See-Yong *et al*. [19] reported red boil disease affecting cultured estuary grouper (*E. salmoides*) in floating cages in the Straits of Penang, Malaysia. Based on colony morphology on selective media and pathogenicity, they concluded that *V. parahaemolyticus* is the causative agent. The diseased fish were found darker in color with hemorrhagic abscesses. In addition, they reported that most cases were asymptomatic due to an acute course of infection. Another case involving greasy grouper (*Epinephelus malabaricus*), silver seabass (*L. calcarifer*), and golden snapper (*L. johni*) were formerly reported by Wong and Leong [91] affecting floating cages area in Penang and Perak, the northern region of Peninsular Malaysia. They found that Group I (consist of *V. parahaemolyticus, V. alginolyticus, V. harveyi*, and *V. vulnificus*) with 3 fish species, greasy grouper, silver seabass, and golden snapper predominately with 42%, 56%, and 62% prevalence, respectively.

Moreover, vibriosis outbreaks are not limited to Peninsular Malaysia but rather spread across Borneo, East Malaysia. For example, Ransangan and Mustafa [92] reported a vibriosis outbreak of Asian seabass cultured in net cages in Sabah, Malaysia, in February 2008. Their work revealed that *V. harveyi* is the causative pathogen. The fish were characterized by deep skin and fin ulceration, dark pigmentation, lack of appetite, ascites in the body cavity, and enlarged liver and spleen. Further analysis discovered that the isolated *V. harveyi* displayed high virulence with a median lethal dose (LD_50_) of 1.4×10^4^ CFU g^-1^ in Asian seabass and 8.33×10^3^ CFU g^-1^ in humpback grouper [93].

Recently, in September 2016, a concurrent infection by *V. harveyi* and *V. alginolyticus* was reported affecting cultured hybrid groupers reared in marine floating cage-culture of Selangor, Malaysia [94]. Within 10 days, 29% of the farm’s juvenile groupers died. Clinical symptoms and signs such as lethargy, increased mucus secretion, rotting fins, liver and kidney congestion, and spleen enlargement were all seen in the diseased groupers, whereas generalized congestion of the brains and internal organs was also reported. Interestingly, other cultured fish species produced in the exact location, such as Asian seabass, snapper, and golden pompano, were not harmed. More recently, according to Amalina *et al*. [95], the distribution of *Vibrio* spp. isolated from cultured groupers in Peninsular Malaysia was predominated by *V. vulnificus*, *V. alginolyticus*, and *V. parahaemolyticus* with 33%, 24%, and 22%, respectively. Thus, vibriosis has remained a severe problem in Malaysian cultured marine fish since the 90s, resulting in significant economic losses. Total losses among Malaysia’s three main grouper, snapper, and seabass culture species were USD 7.4 million in 1990 [95].

According to a recent assessment on the management costs of Asian seabass cage culture in Malaysia, the average loss due to vibriosis was calculated to be USD0.24/tail, which can be broken down into mortality of 79.2%, treatment of 20.8%, and diagnosis of 1.25% [96]. As a result, the development of an effective vaccination approach to prevent disease epidemics is desirable.

#### Development of a vaccine against Vibrio harveyi

One of the earliest attempts by local researchers was reported byMohd-Aris *et a*l. [97], who discovered virulence-associated genes, serine protease (VHS), and OMP from pathogenic *V. harveyi*. Using computational prediction, further characterization of these genes revealed that both VHS and OMP are composed of 62 and 36 antigenic sites, potentially developing an effective live-attenuated vaccine candidate. Following success in the characterization of *V. harveyi* vaccine candidates, a live-attenuated vaccine was developed by selective gene deletion of virulence-associated protease and subjected to vaccination trial in tiger grouper [55]. In week 4, a single dose vaccination regime was employed before the fish was challenged with *V. harveyi*. Their work achieved a moderate satisfactory RPS of 52%. At the same time, transcriptomic profiling revealed the upregulation of the autophagosome pathway, coagulation, complement cascade pathways, and antigen processing and presentation pathways. Another trial of the same vaccine was reported by Chin *et al*. [56], embarking on different fish species of Asian seabass, different vaccine routes of bath immersion, and double dose regime, resulting in improved efficacy. The vaccine was administered on day 0 and 14 before the fish were challenged by immersion at 10^7^ CFU/mL of *V. harveyi*. RPS was recorded at 68%, and higher expression (p<0.05) of the chemokine ligand-4 and MHC I genes in the skin and liver of vaccinated fish was noted.

Classical preparation of the killed vaccine has also proved effective in combating *V. harveyi* infection. Abu Nor *et al*. [98] recently reported that double dose injection of formalin-killed whole-cell vaccine of *V. harveyi* was successfully reduced mortality in marine red hybrid tilapia to 16% after the fish were intraperitoneally challenged with 10^8^ CFU/mL of *V. harveyi* at week 4. A significant increment (p<0.05) in IgM serum, mucus, and gut lavage antibody titer and lysozyme activities was achieved as early as 1-week post-vaccination.

#### Development of a vaccine against V. alginolyticus

Omp has long been recognized as a potential vaccine candidate in most Gram-negative bacteria, owning to its highly conserved region across serotypes and virulence attributes [99]. Besides, scholars reported the antigenic sites of OmpK and OmpW of *V. alginolyticus* consisting of 34 and 27 antigenic sites, respectively, and deemed suitable for vaccine candidates [57]. Interestingly, in an experimental trial, juvenile hybrid groupers were injected with inactivated *Escherichia coli* expressing the OmpK, and OmpW resulted in the RPS of 100% and 63%, respectively, after i.p challenged with a high dose (10^9^ CFU/mL) of a virulent strain of *V. alginolyticus* [58]. Besides, the IgM level against OmpK was significantly higher (p<0.05) than OmpW suggesting the superiority of the OmpK recombinant vaccine against *V. alginolyticus* infection.

More recently, recombinant OmpK vaccine versatility in providing cross-protection against different *Vibrio* species of *V. harveyi*, *V. alginolyticus*, and *V. parahaemolyticus* in Asian seabass has been reported [59]. Furthermore, in an experimental trial, a single i.p injection of recombinant OmpK vaccine resulted in 90-100% survival after being challenged with a high concentration of *V. harveyi*, *V. alginolyticus*, and *V. parahaemolyticus*.

## Challenges and Future of Fish Vaccine in Malaysia

### Commercialization of local vaccine

Increase frequencies of emerging and ­re-emerging diseases among aquaculture fish species as witnessed in the past few years, resulting in considerable economic losses. This trend is expected to drive demand for commercial aquaculture vaccines locally and globally. Unfortunately, although local researchers have achieved a lot and massive investment in research grants, there is still no locally produced fish vaccine available in the country. In fact, other veterinary medical treatments for use in farmed aquatic animals are in severely short supply. This scenario is a severe impediment to disease prevention and response, resulting in welfare issues and stifling the growth of Malaysian aquaculture.

The commercialization of vaccines or any research product is a complex process, and there is no one-size-fits-all formula that will ensure its success [100]. It usually entails a lengthy process and requires a hefty investment; both are frequently a source of difficulty for researchers. Aside from these two crucial factors, several scholars have identified several issues that local researchers may confront when commercializing their research output. Lack of entrepreneurial knowledge and skill, the inefficiency of the Technology Transfer Center, the availability of potential licensee, linkage gaps between industry and researchers, limited workforce, and poor marketing strategies are additional challenges in bringing research outputs into the viable market [101-104]. A dynamic framework for effective commercialization of research products was proposed by Ismail *et al*. [100], consisting of eight elements: The researchers’ knowledge, skills, and personal traits, product idea creation, development, packaging, and promotion, commercialization paths, gaining a competitive advantage in the market, selecting business partners, fostering a positive working relationship with business partners, and providing facilities and support.

Another hurdle in commercializing aquaculture vaccines in Malaysia is the limited availability of animal vaccine manufacturing plants. Like any other biological product, vaccines for commercial use should comply with good manufacturing guidelines. However, unlike conventional pharmaceutical goods, vaccine production involves biological processes such as microbe cultivation and extraction from living cells, which sometimes display intrinsic variability [105]. Therefore, stringent protocol at all production stages is applied, which can only be accomplished in a high-tech manufacturing facility. Because of the cost and complexity of the operation, the vast majority of aquaculture vaccine supply currently comes from a handful of developed countries. Despite the fact that Southeast Asian countries produce some of the world’s best fish and fisheries goods [106], Southeast Asian countries venturing into fish vaccine manufacturing is unheard of. Although the alliance is mostly composed of developing nations, establishing a regional aquaculture vaccine supply chain makes sense in terms of collective food security and supply, socioeconomic growth, and the sector’s sustainability. A medium-sized central manufacturing facility and open-system bioreactors will ensure a low-resource environment and simplify the production of aquaculture vaccines against regionally significant fish diseases. Several factors, however, should be considered before committing to this capacity.

### Limited studies on vaccination field trial

Field trials are required to accurately assess a vaccine’s efficacy, safety, and overall performance in real-world settings. In contrast to controlled laboratory circumstances, the dynamic interaction between host, pathogen, and environment in a production setting can result in variances in immune response and vaccine effectiveness [107]. This event is significant for a fish vaccine for at least two reasons: First, site-specific water physiochemical profiles can affect fish immunity and disease occurrence, and second, physical rearing conditions can vary greatly depending on the rearing technology used [108]. As a result, field testing is a mandatory requirement for innovative vaccines intended for commercial use, and regulatory agencies require it prior to granting full authorization. To achieve successful testing, meticulous planning, full cooperation from farm operators, and massive investment are frequently required. Unfortunately, vaccine field trials in Malaysia are insufficient and should be expanded to expedite commercialization. The following sections describe some critical aspects of the fish vaccinology field trial.

#### Establishing the vaccination protocol

Researchers, farm managers, and operators should collaboratively develop a suitable field vaccination protocol. This step is crucial to ensure all parties involved in the study are aware of their roles and responsibilities and the importance of adhering to the protocol. This is because any deviation that occurs throughout the implementation might affect the trial’s outcome. Therefore, investigators should carefully justify any unforeseen scenarios and provide immediate responses to minimize the study outcome’s impact. Besides, the vaccination regime adopted in the field study must be supported by laboratory trial findings, and other practices should be resembling regular activities on the farm. A set of guidelines from the EU commission (EMA/CVMP/IWP/314550/2010) entitled “Guideline on the design of studies to evaluate the safety and efficacy of fish vaccines” is an excellent reference for this purpose.

#### Site selection, epidemiology, and immune response monitoring

The fish will be subjected to the disease’s natural challenge in the field trial. Therefore, when selecting a trial site, researchers should examine the scale of the disease’s spread and the number of new cases. There is a considerable probability of failure in the field vaccination trial due to the absence of the pathogen. The lack of disease outbreaks in the vaccinated fish population will result in insufficient evidence of vaccine efficacy because the challenge pressure remains unknown. Therefore, investigators should conduct important on-farm epidemiological surveillance to ascertain the critical period of infection and disease prevalence before conducting the vaccine trial.

Furthermore, the quality of the raw data collected during the trial is critical. Farm operators should be educated on the value of accurate record-keeping, which reflects the quality of the final product following the trial period. In addition, the use of mortality records between the vaccinated and control groups is frequently insufficient. It should be complemented by routine response monitoring, such as antibody titer, pathogen isolation, side effect score, and assessment of water quality. These parameters must be explicitly established in the study protocol and justified considering the vaccine’s indications. If no outbreak occurs during the trial, this data can be used to provide evidence of the vaccine’s immunological response. This correlate of protection can be used as an alternative outcome for vaccine evaluation [109].

#### Control groups

In most vaccination studies, a group of vaccinated fish is compared to an equivalent group of unvaccinated or placebo fish. The investigator should choose the controls to provide evidence of infection and represent a group of fish to which the vaccinated group can be compared legitimately. Two approaches are most commonly chosen in comparing these groups: (1) Comparison of vaccinated and control groups in the same captivity and (2) comparison between vaccinated and control groups kept in different but identical captivity within a similar farm. The first approach is customarily preferred since both fish groups will be subjected to equivalent conditions and challenges. Individual identification, such as pit tagging or specialized dye, can be used to accomplish this. This approach, however, is only possible for a vaccine that can be administered on an individual fish basis and is unsuitable for oral and immersion treatment. Besides, researchers should also consider the likelihood of the herd immunity effect, which occurs when a population of vaccinated fish becomes immune, making disease transmission to an unvaccinated group improbable.

### Lack of studies on viral vaccine

For decades, R&D on fish vaccines in Malaysia has been concentrated based on bacterial etiology. Although virus disease has been implicated in the local aquaculture industry since the 1990s, it is not the subject of interest by local researchers. This is most likely owing to the country’s inadequate capacity and capability in this subject. Nowadays, only a few laboratories are prepared to maintain fish cell cultures and virus purification. In addition, producing a fish virus vaccine is indeed a very challenging and time-consuming process. The fish virus has many subtypes or serotypes, and vaccines derived from one subtype are typically less effective against other subtypes [110]. In addition, changes or mutations can occur when the virus reproduces rapidly in the infected fish [111], especially with the RNA virus. The mutation process is often disadvantageous for the viruses, but not always since certain viruses become stronger due to the mutation. Thus, in some cases, the previously developed vaccines are less effective or not effective at all against the newly mutated virus [112]. This phenomenon has caused vaccine development to be a never-ending story that consumes a great deal of expense.

### Future of fish vaccines in Malaysia

Malaysia’s aquaculture industry will continue to proliferate while preserving a competitive edge as market demand for fish increases. This tendency is highly correlated with population growth, rapid urbanization, increase in household income, and international trade expansion, all of which contributed to increased fish consumption per capita [113]. Besides, the industry has generated numerous employment and income generation to the locals and ensures national food security and advancing the sustainable fisheries agenda. Continuous industrial growth will reduce dependence on imported fisheries products while easing pressure on wild fish stocks. Regrettably, the aquaculture industry’s rapid rise has been stymied by recurrent infectious disease outbreaks. Vaccination is considered a viable solution and might offer a brighter future to the industry. At present, fish vaccinations in Malaysia are still limited to a few farms and hatcheries. These operators engage in large-scale farming, hence have the financial means to invest in vaccinations.

The active role played by various organizations, including the Department of Fisheries, local fisheries associations, and universities in fish vaccines gaining wider local attention. Other than that, an increment in the public call for healthy (antibiotics-free) food is probably molding Malaysia’s future of fish vaccinations. The use of vaccinations in aquaculture is being considered as a substitute for antimicrobial medications and chemotherapy. In response, the MyAP-AMR for 2017-2021opposed the irresponsible and uncontrol use of antibiotics in the food production industry. Indiscriminate use of antibiotics in aquaculture would greatly influence the environment due to drug residues, affecting water’s natural microbiota and accelerating the development of antibiotic-resistant bacteria.

Furthermore, water contaminated by these untreated aquaculture effluents is recycled into agricultural land, resulting in elevated antibiotic residue levels in groundwater, hence further increasing the risk of antibiotic-resistant microbes. Alarmingly, evidence of multiple resistant bacteria and resistance genes has been previously reported in Malaysian aquaculture and its environs [114-116]. Vaccines, unlike antibiotics, are widely accepted as the safest, practical, and cost-effective way of preventing aquatic animal diseases. Vaccination has been shown to be critical to the success of Norwegian Atlantic salmon farming [117]. To summarize, salmon farming in Norway suffered significant losses in the 1980s because of salmonid *Vibrio* infections and furunculosis, and farmers relied heavily on antibiotics to battle the diseases. In response, a salmonid vibriosis immersion vaccine and an injectable *Aeromonas salmonicida* vaccine were developed and applied in the farms [118-120]. The vaccines’ high effectiveness has successfully controlled the disease and immediately resulted in a decline of antibiotic dependence [121]. Therefore, we believe that the mass application of vaccines in the local aquaculture industry is beneficial by increasing the industry’s resilience. On the other hand, a thorough analysis of the prospective market and sales of local fish vaccines is highly recommended and should be pursued. Then, with a better grasp of the industry’s needs, competent authorities and local experts can continue their R&D of fish vaccines.

## Conclusion

The Malaysian government has licensed three aquatic vaccination products thus far, all of which are imported. Despite the encouraging milestones achieved by scholars, there are currently no locally produced fish vaccines accessible to protect against various economically relevant diseases. Currently, no local company manufactures aquaculture vaccines due to the complexity and high cost of development and manufacturing, the lengthy registration process, and inadequate scientific and technical competence. Therefore, the Malaysian government’s continued support for businesses venturing into the aquatic vaccine manufacturing industry is crucial. For instance, the Malaysian government has launched the Malaysia Grand Challenge to promote research, development, commercialization, and innovation. These incentives are accessible to start-ups, small-and medium-enterprises, multinational corporations, and individuals moving into new areas. Besides, the development and commercialization of fish vaccines should be collaborative between researchers and private companies from the beginning of the process. We believed that successfully commercializing a local vaccine would spur the development of additional innovative fish vaccines against economically significant diseases such as vibriosis, streptococcosis, viral nervous necrosis, iridovirus disease, and TiLV would eventually benefit the aquaculture industry in the long run.

## Authors’ Contributions

MSMR and MF: Conceived and framed the main idea of this manuscript. MSMR: Prepared the first draft. The first draft was read, criticized, and corrected by AA, RR, NNM, NR and MF. MSMR and MF proofread the second draft and finalized the manuscript. All authors read and approved the final manuscript.
